# Isolated Hepatic Tuberculosis Masquerading as Hepatic Cystadenoma: A rare presentation

**DOI:** 10.12669/pjms.42.(11AASC).15660

**Published:** 2026-04

**Authors:** Laiba Khalid, Abdullah bin Faisal, Asfia Arham Khursheed, Muhammad Rizwan Khan

**Affiliations:** 1 Laiba Khalid, Medical College, Aga Khan University Hospital, Karachi, Pakistan; 2 Abdullah bin Faisal, Medical College, Aga Khan University Hospital, Karachi, Pakistan; 3 Asfia Arham Khursheed, FCPS, Department of Surgery, Aga Khan University Hospital, Karachi, Pakistan; 4 Muhammad Rizwan Khan, FRCS, Department of Surgery, Aga Khan University Hospital, Karachi, Pakistan

**Keywords:** Diagnostic Imaging, Extrapulmonary Tuberculosis, Granulomatous Inflammation, Hepatic Tuberculosis, Hepatic Cystadenoma

## Abstract

**Background:**

Tuberculosis (TB) remains a global health issue, with an estimated 10 million new cases reported in 2019. While pulmonary TB is prevalent, extrapulmonary TB, including hepatic tuberculosis, poses significant challenges, particularly in immunocompromised individuals. Hepatic tuberculosis is rare, constituting about 3% of extrapulmonary TB cases and is often diagnosed in the context of miliary TB. Its diagnosis is complicated by non-specific symptoms and difficulties in differentiating it from other hepatic lesions on imaging.

**Case:**

A 46 years old female presented with moderate upper abdominal pain and weight loss over six months, with no gastrointestinal symptoms. Initial evaluations, including liver function tests and imaging, indicated cholelithiasis and a cystic hepatic lesion. A triphasic CT scan suggested a hepatic cystadenoma. Given the symptoms and the potential malignancy of the lesion, surgical resection was performed, revealing granulomatous inflammation on histopathology, confirming isolated hepatic tuberculosis. The patient was treated with a standard anti-TB regimen and showed significant improvement.

**Conclusion:**

Isolated hepatic tuberculosis can mimic malignancies such as hepatic cystadenoma, complicating diagnosis and treatment. Awareness of this rare presentation is crucial, especially in TB-endemic regions, to prevent misdiagnosis and unnecessary interventions. Early recognition and appropriate anti-TB therapy can lead to favorable outcomes, as demonstrated in this case.

## INTRODUCTION

Tuberculosis (TB) is a global health problem, with an estimated ten million people falling ill with TB in 2019.^1^ While pulmonary tuberculosis is the most common form, extrapulmonary tuberculosis including hepatic tuberculosis, remains a significant health concern especially in the immunocompromised individuals. Extrapulmonary tuberculosis (EPTB) has a reported cure rate of 40.7%, mainly due to lack of clear guidelines for diagnosis and treatment of disease leading to delayed intervention and disease progression.^2^ Hepatic tuberculosis is a rare entity accounting for 3% of all EPTB and is mostly diagnosed as a part of miliary TB. Isolated hepatic TB has been seldom reported in literature. It is often a clinical and radiological dilemma and is misdiagnosed due to its non-specific symptoms and the difficulty in distinguishing it from other hepatic lesions on imaging.^3^

Our case provides an insight into this rare disease with atypical presentation and clinical features including the complexity of radiological findings masquerading as a hepatic cystadenoma.

## CASE PRESENTATION

A 46 years old female presented to our clinic with moderate intensity upper abdominal pain for three months. She had a history of weight loss of approximately 3-4kg over the past six months but reported no other gastrointestinal or systemic symptoms. Her viral markers were positive for previous Hepatitis B infection. Further blood tests revealed an albumin level of 4.1g/dL (normal range: 35-5.2g/dL), elevated AST of 135 IU/L (normal range: <31IU/L), and GGT of 102 IU/L (normal range: <38IU/L). Additional labs including blood profile, chemistry profile and total bilirubin levels were within normal limits. Ultrasound of liver and gallbladder showed cholelithiasis and a cystic lesion in liver. Alpha fetoprotein levels were normal and the serology for amoebiasis and echinococcus were negative.

**Fig.1:**
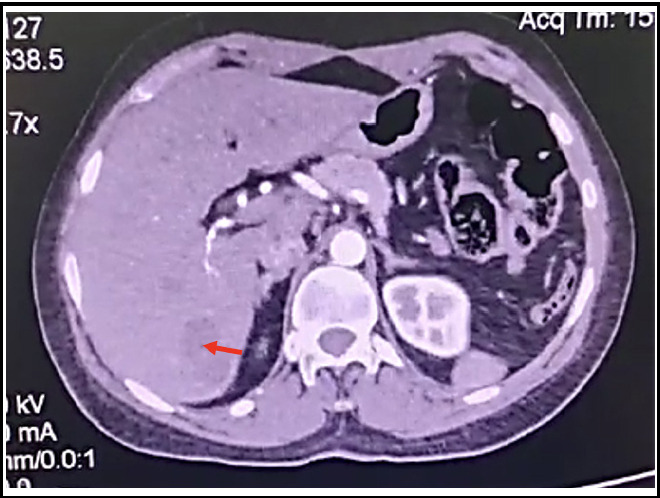
Axial section of CT scan showing bilobed cystic lesion in segment VII (red arrow).

A triphasic CT scan was done for further evaluation, which revealed bilobed cystic lesion in hepatic segment VII without arterial enhancement or washout as shown in Figure 1. A small biliary channel extending from the porta hepatis to the cystic structure was observed, and a radiological diagnosis of cystadenoma was made.

Given her clinical symptoms and the possible premalignant nature of the diagnosis, the patient was advised surgical resection of the liver lesion. The abdomen was entered via a subcostal incision. The right lobe of liver was mobilized and location of the lesion was reconfirmed with intraoperative ultrasound. Transection margins were marked approximately 1cm away from the tumor. Dissection of the liver parenchyma was done using Cavitron Ultrasonic Surgical Aspirator (CUSA) and harmonic scalpel, primarily focusing on segment VII. The branch of the posterior portal vein supplying segment VII was ligated. A non-anatomical right posterior segmentectomy was done along with the cholecystectomy.

Following the procedure, the patient was admitted to a special care unit in a stable condition and followed standard recovery protocol. She did well postoperatively and was discharged on post-operative day four. On subsequent follow-up, final histopathology showed evidence of granulomatous inflammation suggestive of Tuberculosis. She was referred to infectious diseases for further management. The patient was prescribed a combination of rifampicin 150 mg, isoniazid 75 mg, pyrazinamide 400 mg and ethambutol hydrochloride 275 mg for nine months. After completing treatment, she followed up in clinic with no active complaints and is clinically doing well.

## DISCUSSION

Isolated hepatic TB is a rare form of mycobacterium infection that presents significant diagnostic challenges due to its non-specific clinical, laboratory, and imaging features.^2^ In 2020, according to a World Health Organization (WHO) report, 573,000 individuals in Pakistan developed TB out of which 46,000 patients died.^4^ However, the precise number of isolated hepatic TB cases is not readily available, underlining the infrequency and possible under diagnosis of this condition.

Patients with hepatic TB often present with constitutional symptoms (72%), abdominal pain (58%), and fever (28%). Physical examination may reveal hepatomegaly (51%). Laboratory findings often include elevated alkaline phosphatase (79%), elevated aminotransferases (42%), and hypoalbuminemia (45%).^5^ Our patient presented with abdominal pain. However, fever, which is a classical sign of, TB was not present.

Key imaging modalities for TB include chest radiography, CT, and MRI. On imaging, hepatic TB is commonly detected as multiple space-occupying lesions. Additionally, the presence of calcifications and a pseudocapsule may favor a diagnosis of hepatic TB.^5^,^6^ In a study of 103 patients, the commonest presentation of extra-pulmonary TB was lymphadenopathy, followed by pleural and bone disease. Extra-pulmonary TB was diagnosed by biopsy or excision of the extra-pulmonary site.^6^ Our patient underwent a triphasic CT scan which reported findings suggestive of cystadenoma. Due to the location and cystic nature of the lesion, we opted for upfront resection as biopsy was technically difficult.

Previous studies on isolated hepatic TB show that most cases remain undiagnosed preoperatively as acid-fast stain and PCR positivity are uncommon findings. Diagnosis is usually based on final histopathological analysis which shows granulomatous inflammation in 97.5% of patients with, Langhan’s giant cells in 95% and caseous necrosis in 85%.^7^ The WHO recommendations for diagnosis of extra-pulmonary TB include obtaining a sample for culture from the extra-pulmonary site and suggest examination of a CXR and sputum cultures to identify patients with simultaneous pulmonary involvement.^6^

The differential diagnosis of isolated hepatic TB includes conditions such as metastasis, lymphoproliferative diseases, and other granulomatous conditions like sarcoidosis and fungal infections. Conversely, hepatic cystadenomas are uncommon benign cystic neoplasms of the liver. They occur predominantly in middle-aged patients and are more common in women. Although biliary cystadenomas are benign tumors, they may recur after excision and have the potential for malignant degeneration into biliary cystadenocarcinomas.^8^

Misdiagnosis can occur due to the disease’s ability to mimic these conditions, particularly in its disseminated form. In one case series, isolated hepatic TB was provisionally diagnosed as lymphoma due to its unusual and restricted involvement of the liver and spleen.^9^ The correct diagnosis of isolated hepatic TB is crucial, especially in patients with underlying TB. Incorrect diagnosis can lead to inappropriate treatment and potentially fatal outcomes. In a case series in East London, the time between symptom onset and diagnosis was found to be unacceptably high, more than twice that of lung TB.^10^

Treatment with standard anti-TB therapy can effectively manage isolated hepatic TB and may be more beneficial to the patient if treatment is initiated soon after onset of symptoms. In our patient, we administered standard anti-TB medication, which led to the resolution of the underlying problem.

## CONCLUSION

Isolated hepatic TB is a rare condition that can mimic a malignancy clinico-radiologically. Calcifications and pseudo-capsule appearance on multiphase CT scan may help in differentiating isolated hepatic TB from hepatic malignancy. This case report highlights the importance of considering isolated hepatic TB in the differential diagnosis dealing with hepatic lesions, especially in regions where tuberculosis is endemic. Definitive testing including cultures from extra-hepatic sites, and tissue biopsy may be imperative for diagnosis. Early diagnosis and appropriate treatment can prevent unnecessary interventions and improve patient outcomes. Our patient’s case is a prime example of how hepatic tuberculosis can mimic as hepatic cystadenoma, leading to a delay in the correct diagnosis and treatment. However, with the correct diagnosis and appropriate treatment, the patient’s condition significantly improved.

### Authors’ contributions:

**LK AF:** Data collection, interpretation and manuscript writing.

**AAK:** Conceptualization, Data interpretation, manuscript review and responsible for the accuracy of the work study.

**MRK:** Conceptualization, manuscript review.
